# Duchenne Regulatory Science Consortium Meeting on Disease Progression Modeling for Duchenne Muscular Dystrophy

**DOI:** 10.1371/currents.md.83071bbd728982f2f1073f4950e03586

**Published:** 2017-01-12

**Authors:** Jane Larkindale, Richard Abresch, Enrique Aviles, Abby Bronson, Janice Chin, Pat Furlong, Heather Gordish-Dressman, Elizabeth Habeeb-Louks, Erik Henricson, Hans Kroger, Charles Lynn, Stephen Lynn, Dana Martin, Glen Nuckolls, William Rooney, Klaus Romero, Lee Sweeney, Krista Vandenborne, Glenn Walter, Jodi Wolff, Brenda Wong, Craig M. McDonald, members of the Duchenne Regulatory Science Consortium, Imaging-DMD Consortium and the CINRG Investigators

**Affiliations:** Department of Physical Medicine and Rehabilitation, University of California, Sacramento, California, USA; Duchenne Regulatory Science Consortium, Critical Path Institute, Tucson, Arizona, USA; Parent Project Muscular Dystrophy, Hackensack, New Jersey, USA; Pfizer, Rare Disease Research Unit, Cambridge, Massachusetts, USA; Parent Project Muscular Dystrophy, Hackensack, New Jersey, USA; Children's National Medical Center, Research Center for Genetic Medicine, Washington, DC, USA; Parent Project Muscular Dystrophy, Hackensack, New Jersey, USA; University of California Davis Medical Center, Department of Physical Medicine and Rehabilitation, Sacramento, California, USA; PTC Therapeutics, South Plainfield, New Jersey, USA; Duchenne Regulatory Science Consortium, Critical Path Institute, Tucson, Arizona, USA; Stephen Lynn, Newcastle University, Newcastle upon Tyne, UK; Sarepta Therapeutics, Medical Affairs & Patient Advocacy, Cambridge, Massachusetts, USA; NINDS, National Institute of Health, Washington DC, USA; Advanced Imaging Research Center, Oregon Health & Science University, Portland, Oregon, USA; Duchenne Regulatory Science Consortium, Critical Path Institute, Tucson, Arizona, USA; Department of Pharmacology and Therapeutics, University of Florida, Gainesville, Florida, USA; Department of Physical Therapy, University of Florida, Gainesville, Florida, USA; Department of Physiology and Functional Genomics, University of Florida, Gainesville, Florida, USA; Santhera Pharmaceuticals, Tucson, Arizona, USA; Division of Pediatric Neurology, Cincinnati Children's Hospital Medical Center, Cincinnati, Ohio, USA; Department of Physical Medicine and Rehabilitation, University of California, Sacramento, California, USA

## Abstract

Introduction: The Duchenne Regulatory Science Consortium (D-RSC) was established to develop tools to accelerate drug development for DMD.  The resulting tools are anticipated to meet validity requirements outlined by qualification/endorsement pathways at both the U.S. Food and Drug Administration (FDA) and European Medicines Administration (EMA), and will be made available to the drug development community. The initial goals of the consortium include the development of a disease progression model, with the goal of creating a model that would be used to forecast changes in clinically meaningful endpoints, which would inform clinical trial protocol development and data analysis.

Methods: In April of 2016 the consortium and other experts met to formulate plans for the development of the model.

Conclusions: Here we report the results of the meeting, and discussion as to the form of the model that we plan to move forward to develop, after input from the regulatory authorities.

## Introduction

Recent advances in research for Duchenne Muscular Dystrophy (DMD) have resulted in a robust pipeline of drugs in development to treat the disease, with 23 interventional trials ongoing as of June 1, 2016 (clinicaltrials.gov). The prevalence of DMD is approximately 1.38 per 10,000 male individuals aged 5 to 24 years^1^, and given the resulting small numbers of patients with DMD, recruiting new trials with larger numbers of patients will be problematic. This issue is compounded by the fact that most trials to date have focused on endpoints that require the patient to be ambulatory, and that many test articles are directed toward prognostically enriched sub-populations of patients with specific genetic and functional characteristics, thus excluding large parts of the population. In light of the resulting need to design and conduct smaller, more targeted clinical trials, regulatory authorities are open to considering novel endpoints to look at efficacy of such drugs, so long as such endpoints are scientifically justified (FDA and EMA guidances)^2^^,^ 3.

The most advanced potential therapeutics are now completing late stage trials and are being proposed to the regulatory authorities for approval. However, to date only one late stage trial (Santhera’s Idebenone) has met its pre-specified primary endpoint. Different therapeutic mechanisms produce different expectations for the magnitude and time course of a treatment effect. Therapeutics that provide a transient improvement of function may be studied in for shorter durations. Therapeutics that stabilize disease progression require longer study durations to demonstrate slowing of disease progression, particularly in young patients who may be acquiring skills at a slower rate than typically developing unaffected children. Pre-specified and post-hoc subgroup analyses, comparisons to historical natural history data and analyses of specific endpoints most sensitive to a treatment effect in a short duration 12-month trial do, however, indicate the possibility of a drug effect in some cases. The interpretation of such analyses is an ongoing point of discussion throughout the community and with the regulatory authorities. It is clear that in order to conclusively demonstrate that drugs are effective, we need a clearer understanding of sources of variability in disease progression of DMD patients, so that appropriate endpoints can be investigated in appropriately selected patient subgroups. This should allow smaller, shorter trials to be informative.

The Duchenne Regulatory Science Consortium (D-RSC) was established to develop tools to accelerate drug development for DMD. The first tool that will be developed by the consortium is a disease progression model. The resulting tools are anticipated to meet validity requirements outlined by the fit-for-purpose pathway at the U.S. Food and Drug Administration (FDA) and the Qualification of Novel Methodologies for Medicine Development pathway at European Medicines Administration (EMA), and will be made available to the drug development community. The tools must meet the criteria of being clinically meaningful, useful to drug developers and acceptable to the regulatory authorities. To achieve this, the D-RSC is creating an aggregated clinical dataset from multiple industry and academic sources. The data will be used in the first instance to create a disease progression model that will describe how the disease progresses in subgroups of patients defined by clinical variables, with the initial goal of informing trial inclusion criteria and endpoint selection. If the model is formally endorsed by regulatory authorities as is anticipated, it is a further goal of D-RSC to make the model broadly available to the Duchenne research community. D-RSC members and clinical experts held an inaugural meeting in April of 2016 to discuss group practices and development of the initial model.

**Summary of discussion of Duchenne disease progression:**

Clinical experts lead the discussion with a summary of analyses from existing datasets, highlighting how different endpoints change over the disease course, how endpoints correlate with each other and how they could be used for modeling disease progression. Dr. Craig McDonald discussed data from the UC Davis / Cooperative International Neuromuscular Research Group (CINRG) Duchenne Natural History Study (DNHS)^4^ and from clinical trials, showing the changes in functional and respiratory endpoints over the course of the disease. He showed that, although progression of disease is variable between patients, it follows a predictable pattern of loss of specific functional milestones (ability to rise from the floor, ability to climb stairs, ability to walk, loss of upper limb function, and loss of respiratory function etc.)^5^. He presented data demonstrating that although measurements of the length of time it takes to complete tasks change over time, loss of functional milestones tends to happen precipitously, with limited change in timed functional tests prior to the sudden loss of the related milestone. Some of the precipitous decline in lower extremity endpoints is due to the onset of a critical threshold of lower extremity loss of muscle fibers (muscle substrate for the targeted therapeutic) in key muscle groups such as the knee extensors or proximal pelvic girdle. The loss of function may also correlate with an event, such as a fracture, that prevents the patient from being able to complete the test. However, the age or the timing of loss of each milestone could be predicted to some extent by the age or time of loss of previous milestones (unpublished data).

In addition, Dr. McDonald showed data demonstrating that functional endpoints are frequently predictive of each other, such that patients that lose a specific milestone function at a given time can be predicted to lose an additional milestone ability within a given time. For example, the age of loss of standing ability correlates with the age of loss of 4-stair climb and ambulation in individual patients, and a baseline time to stand from supine predicts a loss of standing ability, stair climbing ability and ambulation within a defined period of time^6^. Similarly, patients walking less than 300 m in a six minute walk test have more heterogeneity in their change in 6-minute walk distance (6MWD) are much more likely to lose the ability to complete the test within the next year than patients outside of that window^7^. Essentially no patients with baseline 6MWD above 400 meters lose ambulation in 12 months and these patients tend to show little decline in 6MWD over the course of a year. These correlations between milestones are seen throughout the disease, including non-ambulatory endpoints such as loss of ability to self-feed (bring the hand to mouth) and respiratory measures such as time to a forced vital capacity of 50% or 30%. Such correlations can be seen in multiple datasets, both in natural history data and in the placebo arms of trials. These correlations suggest that the pattern of progression of loss of these milestones is consistent, and may be able to be predicted by specific baseline characteristics of the patients. If that pattern of progression could be modeled, it might be possible to predict which patients are likely to lose specific milestone functions in a given period of time, and deviation from such a pattern might be indicative of a treatment effect.

The most commonly used composite endpoint scale that incorporates these milestone functional changes is the Northstar Ambulatory Assessment, which is increasingly being used in clinical trials. The non-linearized scale has each ambulatory milestone reduced to a score between 0 and 2 depending on the patient’s ability to complete the test^8^. Dr. McDonald showed data from one trial, where the overall linearized 100 point scale did not show a statistically significant difference between treated and placebo patients. However, the data shows that patients in the drug arm showed fewer relative losses of every function tested (shifts from a 2 or 1 score on the NSAA to 0) across all 17 functions tested using an odd’s ratio (a reduction in lost clinically meaningful milestones). Thus, considerations of losses of milestones or individual functions by calculation of an odd’s ratio appears to be more statistically robust and clinically meaningful than the change in the overall summated or linearized NSAA score. Dr. McDonald suggested that by summing the scale across all 17 functions or linearizing the scale there may be lost granularity or sensitivity of a scale that can potentially be used across patients across a wide spectrum of ambulatory disease progression.

Dr. McDonald showed data from the CINRG dataset relative to respiratory measures, specifically forced vital capacity (FVC). While absolute forced vital capacity increases with maturation and growth to a plateau phase during adolescence, percent predicted FVC does change in a more linear fashion with disease progression, slowly decreasing from the time patients are quite young. Differences in % predicted FVC can be seen between patients who have taken steroids and those who have not, indicating that the measure might be able to detect a treatment effect^9^. However, Dr. McDonald pointed out challenges with how the community calculates percentage predicted FVC and other spirometry values, based on the boy’s height, which is confounded by steroid treatment. Prediction equations for height based on ulnar length can be used to calculate percent predicted spirometry values. He also noted that it is unclear what a clinically meaningful change in FVC would be in earlier stages in disease. He suggested that passing certain thresholds of FVC, such as 50% predicted FVC (a time when mechanical cough assistance may be recommended) or 30% predicted FVC (when all patients are recommended to have started nocturnal ventilation), might be used as later stage functional milestones in disease, similar to the functional milestones he suggested in earlier stage disease. Alternatively, a threshold of less than 1 liter absolute forced vital capacity has been linked to 5-year survival in DMD and this could be another threshold for time to event analyses later in the disease course.

Dr. Erik Henricson discussed how the functional milestones discussed by Dr. McDonald correlate with patient reported functional health outcomes, and how such outcomes change over the course of disease. Correlation between patient reported outcomes and loss of functional endpoints may be useful to establish the clinical meaningfulness of such measures. He showed that the Pediatric Outcomes Data Collection Instrument (PODCI) mobility -oriented subdomain scales correlate with functional measures, and could be used across a significant age range of patients^10^. He showed data demonstrating that PODCI scores for the transfer and basic mobility subscores change linearly with 6 minute walk distance^11^. When patients in the CINRG natural history study were divided into groups relative to their loss of functional milestones, these groups of patients showed a distinct pattern of change in the PODCI indices^10^. Steroid treatment was associated with significant differences in the age of patients that fit into each milestone group, indicating a measurable treatment effect. This suggests that patient reported outcome scores in Duchenne do link closely to the loss of functional milestones.

The PODCI scale was developed to assess children with a variety of orthopedic limitations, and it describes DMD patients across most stages of disease. However, the instrument demonstrates ceiling effects in highly functional young children and floor effects in individuals with very advanced disease. To address these issues, Dr. Henricson described his team’s ongoing efforts to combine items from multiple scales into a single mobility construct-oriented PRO mobility assessment, the DMD Lifetime Mobility Scale (DMD-LMS) (Erik Henricson, unpublished data). The instrument will include a broadened list of “milestone” tasks that are meaningful to patients (e.g. activities of daily living), including tasks involved in walking and moving, changing and maintaining body position, and lifting and handling objects. Included Items are demonstrated to show responsiveness to differences in disease stage and steroid treatment effects, and are sensitive to 1-year changes in disease progression typical to today’s clinical trial designs. Once complete the DMD-LMS, developed using Item Response Theory techniques, will be a continuous scale instrument that describes mobility-related functional task ability from early ambulatory to late non-ambulatory levels of disease involvement.

Dr. William Rooney discussed data collected by the Imaging-DMD consortium (http://www.imagingdmd.org/), which is looking at skeletal muscle magnetic resonance imaging (MRI) and magnetic resonance spectroscopy (MRS) as measures of disease progression across the course of the disease. The Imaging-DMD consortium was set up to investigate the hypothesis that magnetic resonance (MR) markers of muscle pathology are sensitive to disease progression in boys with DMD across a wide range of disease stages and predictive of loss of function^12^^,^ 13. Dr. Rooney shared data showing the visible changes in fat fraction over time in MR images, which start prior to any loss of function^14^. Dr. Rooney showed images and data that demonstrate that fat fraction (measured by either MRI and MRS) and MRI T2 increase markedly with DMD disease progression. Fat fraction measured by each technique provided different information, but the pattern of increase correlated between the measures^14^.

Imaging DMD has studied 133 patients with DMD and 50 controls over 5 years. Dr. Rooney showed that over time, the fat fraction of the muscle increased in all of the leg muscle groups studied, although the absolute amount of fat detected and the rate of change varied between muscles^13^^,^ 14. Substantial annual changes in fat fraction and MRI T2 values were observed from many muscles of the leg with standardized response means greater than 0.75^14^. He noted that the vastus lateralis muscle could serve as a “sentinel” muscle, showing effects earlier than most functional deficits can be detected, indeed even when functional abilities may be improving developmentally^13^^,^ 14. Other muscles showed later changes in fat fraction, but all muscles tested showed increases over the course of the study.^14^

Dr. Rooney showed data that demonstrated that the fat fraction of the vastus lateralis muscle changes in a Gaussian-type function^15^, which could be modeled statistically based on age and baseline fat fraction. Similar curves could be calculated for the other muscles. The curves mimicked the progression of the disease, suggesting that the model could be used to characterize muscle involvement across disease. The Imaging DMD consortium has additional data that demonstrates that functional endpoints can be mapped to changes in fat fraction in specific muscles based on disease progression modeling (William Rooney and Imaging-DMD Consortium, unpublished data).

**Planning for a disease progression model:**

The group discussed what a useful model of disease progression might look like. For the model to be useful in developing clinical trial protocols, it needs to be able to predict which patients are likely to change in specific endpoints in a statistically meaningful way over a period of less than a couple of years, so as to inform inclusion criteria and size and length of a trial. It needs to be able to predict clinically meaningful changes in the patients, in order to be of use in regulatory decisions. It also needs to be supported by high quality data.

The integrated dataset that D-RSC is building will be limited by the data that have been collected, and that the Duchenne community is willing to share with the consortium. It will integrate multiple datasets so as to include a variety of patients across the disease spectrum and located at different centers so as to represent patients as broadly as possible and account for differences between them. Over time, as more data become available, D-RSC envisages new versions of the model being developed to account for new data that becomes available.

The group agreed that modeling any individual functional endpoint would limit the utility of the model, as only a small proportion of patients are expected to change significantly in any given functional milestone over the period of one year due to disease alone. Measures that could be modeled over longer periods of disease include FVC, Northstar Ambulatory Assessment, patient reported outcome measures such as Dr. Henricson’s longitudinal mobility scale and fat fraction by MRI. Even these measurements do not cover the entire course of disease, but exclude the very young (MRI changes are detected as young as age 4, and patient reported outcomes do not currently cover those younger than 3-4). Late non-ambulatory patients may also not be covered as well by these measures.

The group discussed the use of muscle MR measures as a measurement that changes across disease course, and data supports its relationship with functional changes. Dr. Rooney’s data showed that different muscles showed different patterns of when fat fraction started to increase, and rates of increase, suggesting that fat fraction of different muscles might be best predictive of different functional changes. Dr. Wong noted that the the gluteal muscles are the first to be involved in DMD, and progression of weakness of the gluteal muscles (i.e. pelvic extension) would be expected to correlate with the timed rise from the floor test for early ambulatory DMD patients, while the quadriceps are not yet involved and see less fat infiltration in young DMD boys. Hence MRI of fatty changes of the gluteal muscles could be expected to be informative for disease progression in early stage patients, and might be expected to correlate with specific functional outcomes. Dr. McDonald suggested that creating a combined measurement of fat fraction from multiple muscles might provide a measure to predict multiple functional endpoints across disease.

The group noted that the curves for muscle fat fraction resembled those for manual muscle testing, and wondered how well the fat fraction correlates with muscle strength, both of which have previously been shown to correlate with functional tests^16^. This hypothesis warrants further testing. If the muscle fat fraction changes closely with strength of that muscle, it may be possible to create a relevant composite measure that describes the amount of remaining functional muscle, which might be expected to track closely with functional abilities.

Dr. Klaus Romero suggested that it might be possible to use a variable such as fat fraction, muscle strength or a composite of the two to model across disease stages in order to predict the loss of specific milestones. Loss of carefully selected functional milestones would likely be seen as clinically meaningful by the regulatory authorities, but this would need to be confirmed with FDA/EMA. He suggested that a time-to-event modeling approach could be developed, using the clinically-relevant milestones as the endpoints of interest, once these have been agreed-upon with regulators. This time-to-event model would, in turn, be driven by the parameters derived from non-linear-mixed-effects models for muscle MR (or muscle strength, or a combination), as well as optimized longitudinal outcome measure scales. These non-linear-mixed-effects models would, in turn, capture relevant sources of variability, which were discussed and are described in the following paragraphs. In this framework, the functional milestones would be the ultimate endpoints in a trial, but they would be linked and predicted by quantitative models that describe the progression of continuous optimized scales and biomarkers. The group agreed that this construct would be meaningful to them, and would be likely to be supported by the data.

The disease “milestones” most commonly referred to are functional outcome measures in the ambulatory patient group such as loss of ability to rise from the floor, loss of ability to climb stairs, loss of ability to walk, although upper extremity milestones such as loss of ability to reach overhead or get a hand to the mouth have also been described. There was discussion of additional milestones that could be included to mark changes later in the disease, and it was thought that meeting specific respiratory milestones that correlate with treatment changes, and some of the upper limb scores might be able to be used. It was noted that the definitions of such milestones will need to be carefully agreed-upon, based on a) what is clinically relevant, b) what has been captured in the available data sources, c) what milestones appropriately represent functions across the entire continuum of disease, and d) what makes sense from a drug development and regulatory perspective. This is critically important, as at different centers the loss of the milestones is interpreted differently. For example, definition of “loss of ambulation” can be based on a clinical evaluator or person-reported outcome of loss of ambulation or full-time wheelchair use, the inability to complete a 6 minute walk test, an inability to complete a 10m walk/run test, or an inability to stand or walk even a single step (on the NSAA). For the purposes of this model, we will need to clearly define what we mean by the milestone and ensure that the data in the database reflects those definitions.

The group noted that in considering the data we will need to describe other sources of variability in the population, such as differences in anthropomorphic characteristics (height, weight), variations in use of steroid regimes and other preventive clinical practices, and underlying differences in patient’s genetic characteristics. These factors, as well as baseline functional measures, are expected to affect the progression of the disease in individuals, and need to be incorporated into the proposed modeling approach, along with any biomarker data that can be accessed. The variables that will be used in the final model will be selected after stringent statistical analyses of the dataset, but expert opinion provided in this meeting allows D-RSC to ensure that datasets including variables that are thought to be of interest are included.

## Conclusions

The group concluded that they were interested in pursuing such a modeling approach based on consideration of muscle fat fraction, timed function tests, muscle strength, as well as optimized scales of function in ambulant and non-ambulant patients, with additional consideration of time to event analyses of specific disease milestones. The variables that are finally included in the model will be dictated by the data – both what data is in the integrated dataset and what early stage analyses of that data tells us about what is most relevant to the final model. The context of use of the model would be to forecast changes in clinically meaningful outcome measures, which would inform clinical trial protocol development with respect to inclusion criteria, endpoints to include, and size and length of trials.

## Data Availability Statement

This report is the proceedings of a meeting, and no new data was reported.

## Competing Interest Statement

The authors have declared that no competing interests exist.

## Corresponding Author

Jane Larkindale: jlarkindale@c-path.org
